# Community referral for presumptive TB in Nigeria: a comparison of four models of active case finding

**DOI:** 10.1186/s12889-016-2769-7

**Published:** 2016-02-23

**Authors:** A. O. Adejumo, B. Azuogu, O. Okorie, O. M. Lawal, O. J. Onazi, M. Gidado, O. J. Daniel, J. C. Okeibunor, E. Klinkenberg, E. M. H. Mitchell

**Affiliations:** Department of Community Health and Primary Health Care, Lagos State University Teaching Hospital, Ikeja, Lagos Nigeria; Department of Community Medicine, Ebonyi State University, Abakaliki, Nigeria; Abia State TB and Leprosy Control Programme, Umuahia, Abia Nigeria; Oyo State TB and Leprosy Control programme, Ibadan, Oyo Nigeria; KNCV/ TB CARE I, Abuja, Nigeria; Department of Community Medicine and Primary Care, Olabisi Onabanjo University Teaching Hospital, Sagamu, Ogun Nigeria; Department of Sociology/Anthropology, University of Nigeria, Nsukka, Enugu Nigeria; KNCV Tuberculosis Foundation, the Hague, The Netherlands; Department of Global Health, Academic Medical Center, Amsterdam Institute of Global Health and Development, Amsterdam, The Netherlands

**Keywords:** Presumptive TB referrals, Active case finding, Community workers, Nigeria

## Abstract

**Background:**

Engagement of communities and civil society organizations is a critical part of the Post-2015 End TB Strategy. Since 2007, many models of community referral have been implemented to boost TB case detection in Nigeria. Yet clear insights into the comparative TB yield from particular approaches have been limited.

**Methods:**

We compared four models of active case finding in three Nigerian states. Data on presumptive TB case referral by community workers (CWs), TB diagnoses among referred clients, active case finding model characteristics, and CWs compensation details for 2012 were obtained from implementers and CWs via interviews and log book review. Self-reported performance data were triangulated against routine surveillance data to assess concordance. Analysis focused on assessing the predictors of presumptive TB referral.

**Results:**

CWs referred 4–22 % of presumptive TB clients tested, and 4–24 % of the total TB cases detected. The annual median referral per CW ranged widely among the models from 1 to 48 clients, with an overall average of 13.4 referrals per CW. The highest median referrals (48 per CW/yr) and mean TB diagnoses (7.1/yr) per CW (H =70.850, *p* < 0.001) was obtained by the model with training supervision, and $80/quarterly payments (Comprehensive Quotas-Oriented model). The model with irregularly supervised, trained, and compensated CWs contributed the least to TB case detection with a median of 13 referrals per CW/yr and mean of 0.53 TB diagnoses per CW/yr. Hours spent weekly on presumptive TB referral made the strongest unique contribution (Beta = 0.514, *p* < 0.001) to explaining presumptive TB referral after controlling for other variables.

**Conclusion:**

All community based TB case-finding projects studied referred a relative low number of symptomatic individuals. The study shows that incentivized referral, appropriate selection of CWs, supportive supervision, leveraged treatment support roles, and a responsive TB program to receive clients for testing were the key drivers of community TB case finding.

## Background

Tuberculosis (TB) still constitutes a serious public health problem in Nigeria, despite the implementation of the directly observed treatment short course (DOTS) strategy since 1993, adoption of the World Health Organization (WHO) Stop TB strategy in 2006 and availability of DOTS facilities in all the Local Government Areas (LGAs) in the country [1]. Accessibility of DOTS services is still not sufficient as the majority of the population live some distance from the DOTS centres and most TB diagnostic and treatment centers are located in urban areas. Stigma, poor knowledge of TB, cultural beliefs contribute to poor health seeking behavior, delay or failure to access the health services [1–4]. The TB burden in Nigeria is one of the highest in the world (318 per 100,000) [5] but the case detection rate (16 %) is one of the lowest in the world [6]. Innovative interventions to increase case detection are therefore urgently needed.

Although community participation in health care is an old concept [1] TB control programs have been slower than other disease programs to embrace the role of non-clinicians in detection, care and treatment [7]. However recent studies in high TB burden countries have shown that involvement of community workers (CWs) can improve case notification and/or treatment success [8–12]. Community involvement in TB is part of the STOP TB strategy launched in 2006 to enhance DOTS expansion and reduce the global burden of TB. Implementation of community tuberculosis care (CTBC) in Nigeria commenced in 2007. Currently, CTBC is established in 27 out of 36 states including the federal capital territory (Abuja) [1]. In order to fund CTBC activities in the country, the National Tuberculosis and Leprosy Control Programme (NTBLCP) embraced collaboration with implementing partners. Each partner worked in designated LGAs in each state. Except for Lagos state where three different CTBC implementing partners operate, all other states have only one CTBC implementing partner. Many of the implementing partners work through Community Based Organizations (CBOs) in the selection, training and supervision of the CWs. In addition to referral of presumptive TB cases to the DOTS centres, in some models CWs also serve as treatment supporters and help track those lost to follow up. Each implementing partner adopted a slightly different approach (which we refer to as models) in their CTBC operations. The models vary in mode of recruitment, frequency and method of supervision and motivation of workers, record keeping, level and regularity of compensation, and whether referral quotas were explicitly set (Tables 1 and 2).

**Table 1:** Selection criteria for community workers in the different models

Models	State	Contact person/institution	Who does the selection	Criteria for selection	TB local government supervisor awareness	CBO influence on selection
Direct dealing	Abia	The traditional leader	The traditional leader	1. Known to the traditional leader	The local government TB supervisor was aware but had no influence on the selection process	Does not work with CBOs in their operations
				2. Live in the community		
				3. Respected member of the community		
				4. Previous active involvement in volunteer work was an added advantage		
Supervision without quotas	Oyo	The Medical Officer of health for the local government.	The local government council officials	Involvement in other volunteer work in the local government	The local government TB supervisor was aware but has no influence on the selection process	No influence they had to work with what was provided
Comprehensive quotas-oriented	Lagos	The Community Development Council (CDC)	The CDC but the local government TB supervisor and the Ward health committee made inputs	Involvement in other volunteer work in the local government	The local government TB supervisor was aware and could influence the selection process	No influence they had to work with what was provided
Unsupervised volunteer	Lagos	The Community Development Association (CDA)	The CBO	Any interested member of the community	Not aware of the selection	They determined who was selected

**Table 2:** Comparison of supervision, compensation, and quotas in community TB referral programs

Type of model	Supervision of CWs	Other role of CWs apart from referral	Monetary compensation	Regularity of compensation	Quotas	Record keeping of referrals
Direct dealing	Monthly meeting with TB Local Government Supervisor (TBLS), No field supervision	Optional	$10 quarterly	Not regular	No set quotas	Good
Supervision without quotas	Quarterly meeting with the CBO	Optional	$ 40 quarterly	Fairly regular	No set quotas, however compensation could be suspended if no referrals for 3 consecutive months	Very keen on record keeping
	Irregular field supervision					
Comprehensive quotas-oriented	Monthly meeting with the CBO.	Also serve as treatment supporter for any TB case arising from their referral and also track patient lost to follow up	$ 80 quarterly	Fairly regular	Five presumptive TB referrals every month and 1 TB case	Good record keeping
	Regular field supervision					
unsupervised volunteer	Irregular meetings	Never	Not fixed, any amount between $13.3 and $20 quarterly	Never regular	No quotas	Very poor,
	No field supervision of CWs					

Note: 150 Naira to $1 used for conversion of Nigerian Naira to US dollars

NB: The CWs of the quotas oriented model do not get compensation for a referred case or TB case detected but only get compensation per time period just like CWs of other model

To the best of our knowledge, no study has been conducted in Nigeria to investigate the performance of CWs under the different models in detection of TB cases. We therefore compared four models of enhanced TB case finding and the factors driving differential performance.

## Methods

### Study setting

The coordination of TB control throughout the country is the responsibility of the NTBLCP. TB control is structured along the three tiers of government, i.e. federal, state and local government (district) level. At the national level the NTBLCP is responsible for facilitating policy development, as well as tertiary care, resource mobilization, program evaluation, human resource development, and technical support to state programs. At the state level, the day-to-day program implementation and supervision are carried out by the state TB control officer(s), supported by the state TB supervisors [13]. The state TB programs coordinate TB activities in their respective states and provide secondary care and technical assistance to the LGA level. The LGA is the operational level of the NTBLCP. At this level, TB control activities are the responsibility of local government TB supervisors. Primary health care (PHC) workers carry out TB activities in close collaboration with their respective communities.

The study was conducted from July to December 2013 in the part of the country with highest TB notification, which included two states (Lagos and Oyo) in the South West and one State (Abia) in the South East Lagos State is the commercial center of the country. It has an estimated population of 9 million about 5 % of the national population. Abia and Oyo states have populations of 3.9 million and 5.6 million respectively according to the 2006 national census. Lagos state had three different CTBC models operational, while Oyo and Abia each had one CTBC model.

### Study design

**Fig. 1:**
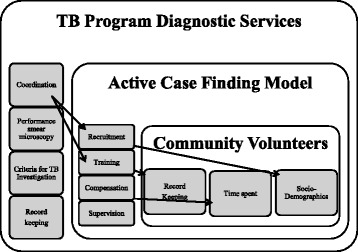
Conceptual framework of CTBC model in Nigeria

The study was designed to evaluate CWs performance, and specifically the influence of socio demographic characteristics, modes of recruitment, supervision, record keeping, level and regularity of compensation, referral quotas, and length of CW training (Fig. 1). To compare the performance of the different CW models, data for 2012 were abstracted from CWs referral registers, CBO registers, health facility ‘suspect’ registers, and TB treatment registers. Structured interviews with CW, implementing partners and CBOs as well as LGA-level TB officers were conducted to obtain details of the models. Triangulation among sources was done to assess agreement.

### Selection of study sites, community-based intervention models, and participants

We purposefully selected different community case finding models in order to ensure diversity of approaches to training, supervision, and compensation schemes. Implementers of four out of five models selected, consented to be included. Selection of local government areas (LGA) where community workers of different partners operated was necessarily opportunistic but co-location in a state ensured comparable policy and reporting frameworks. The four included models are funded by the major tuberculosis donors in Nigeria including TB REACH (World Health Organization/Canadian International Development Agency), United State Agency for International Development (USAID) and The Global Fund for TB, HIV and Malaria. The implementation of the four models was coordinated by different national and international NGOs, i.e. FHI360, AIDS Prevention Initiative Nigeria (APIN), German Leprosy and Tuberculosis Relief Association (GLRA), and KNCV Tuberculosis Foundation. All the CWs and CBOs in the selected LGAs who consented to participate in the study were recruited.

The first model, labelled **‘Direct Dealing’,** did not recruit CWs through CBOs but rather through traditional leaders. The TB supervisors in the LGA supervised the CWs through monthly meetings. No quotas were given to CWs and record keeping was compulsory. CWs were only involved in referral of presumptive TB cases.

In the second referral model, dubbed **“Supervision without Quotas**”, the CBO’s were involved in the recruitment and supervision of the CWs through regular quarterly meetings. Compensation was regularly provided. No quotas were set for the CWs but compensation could be withdrawn if no referral was made in three months.

The third model referred to as **“Comprehensive Quotas-Oriented”** also involved the CBOs in the recruitment and supervision of the CWs through monthly meetings and regular field supervision. In this model CWs also served as treatment supporters of TB patients diagnosed. They were also expected to track TB patients lost to follow up. Workers were expected to refer a minimum of five presumptive TB cases per month and to detect one TB case per month. Referral was not incentivized, i.e. the compensation did not reflect quantity of referrals.

A fourth model, named **“Unsupervised Volunteer”** involved the CBO’s in the recruitment and supervision of the CWs. However, supervision was sporadic, no referral quotas were set, compensation was not specified and payment was highly irregular. The workers seldom saw the supervising CBO and materials for recording presumptive TB referrals were not provided to the CWs.

Except for the Direct Dealing model, the TB LGA supervisors were not involved in the supervision of the CWs (See Tables 1 and 2 below). The CWs were trained for a period of three to five days, depending on the funding partner, using the standard National Community Workers training manual on presumptive TB identification [7]. Each CW was assigned to specific ward within the LGA and with the help of the CBOs, the CWs could create awareness in the community by talking to religious groups/organizations, trade unions and community development associations (CDAs) present within the community. They sometimes go house to house to create awareness of tuberculosis treatment and refer any person with cough for two weeks or more with or without weight loss, fever, tiredness, night sweats, chest pain, shortness of breath or haemoptysis to the nearest DOTS facility. Each arriving presumptive TB client is documented with a referral form which is kept at the DOTS center. The CWs also retain a copy of the referral form (See Table 3). CW could also refer any presumptive TB client outside their catchment area to the nearest DOTS center.

**Table 3:** Comparison of TB case finding performance of four models of community worker referral

Variable	Direct dealing with CWs model (*N* = 15)	Supervision without quotas model (*N* = 60)	Comprehensive quotas oriented model (*N* = 25)	Unsupervised volunteer model (*N* = 24)	Total (*N* = 124)
Annual compensation per CW	$40	$160	$320	≈$80	
Referred clients tested for TB					
Total TB investigations (passive + active)	211	2062	5113	1436	8822
presumptive TB clients referred by the CWs	48	498	1063	56	1665
Proportion of TB investigation due to CW referral	22.7 %	24.2 %	20.8 %	3.9 %	18.9 %
Average presumptive TB clients referred per CW (year)	3.2	8.3	42.5	2.3	13.4
Average CW compensation per referral (year)	$13	$19	$8	$34	
Average CW referrals needed to detect 1 TB case	6	3.3	6	3.1	4.7
TB patients diagnosed					
Total TB patients detected (actively and passively) in LGAs	70	654	1145	413	2282
TB cases from CWs referrals (yr)	8	150	177	18	353
Proportion of the total TB patients contributed by CWs	11.4 %	22.9 %	15.5 %	4.4 %	15.5 %
CW compensation per TB patient diagnosed	$75	$64	$45	$107	
Mean number of TB patients contributed per CW (per year)	0.53	2.5	7.1	0.75	2.8

Note: *n* Number of CW

NB: The number of CWs used in calculation were obtained from the Local government TB register

### Study procedure

In each of the selected LGAs all the CWs were invited through their CBOs or agency to participate in this study. After the purpose and procedures of the research were adequately explained, written informed consent was obtained. A pre-tested semi structured questionnaire was administered to each CW as well as to a representative of the CBOs to obtain information on their mode of recruitment, training and knowledge on presumptive TB identification. In addition, data on the number of presumptive TB cases referred by the CWs and the number of CWs referrals resulting in a TB diagnosis were collected from the CBOs’ registers from January 2012 to December 2013. The data collected from the CBO registers were triangulated with the corresponding figures maintained by the TB surveillance system. In case of discrepancies, the data from the TB program were considered as less biased because it was the data sent to the national TB program and therefore used for analysis. It is common for CWs to refer presumptive TB cases who may not actually reach the heath facility for investigation.

From the questionnaire administered to the CWs, an unweighted TB knowledge score was created from questions about CWs knowledge of 10 elements concerning TB signs and symptoms. The correct answers to each question were scored one mark while each wrong answer was scored zero. Those with a score above and below the median value (of all CWs) were classified as having “good knowledge” and “poor knowledge” of presumptive TB identification respectively.

### Data analysis

The data were analyzed using Statistical Package for Social Sciences (SPSS) version 19. Percentages, mean, median and interquartile range were used to describe the outcome variable. Normality of the distribution was assessed using histogram plot after which a decision was made for the appropriate statistical test. Chi square test and in case of low number of observations Fisher’s exact test were used to determine the association between categorical variables. A Kruskal-Wallis test was used to compare the median values of presumptive TB cases referred across the various models. Mann Whitney *U* test was used to determine the association between knowledge and the median number of presumptive TB cases referred. Spearman Rho correlation was used to determine the correlation between numerical variables. Standard multiple regression was used to assess potential correlation among presumptive TB referrals, duration of engagement as a CW, compensation received and type of CW model to predict presumptive TB referral. Confidence interval was set at 95 % for all statistical tests except otherwise stated. Preliminary analysis was conducted to ensure no violation of the assumption of normality, linearity, multicolinearity and homoscedasticity. Simultaneous or enter method was used in the regression analysis.

### Ethical consideration

Ethical approval for this study was obtained from the ethical committee of the Lagos State University Teaching Hospital. Verbal consent was obtained from the respondents before recruitment into the study and they were assured of strict confidentiality of all the information. Therefore results are not linked to a particular State, nongovernmental organization (NGOs), CBOs, donors, or individuals.

## Results

### Sample characteristics

A total of 115 CWs were recruited into the study. Of these 25 (21.7 %) represented CWs of the Direct Dealing model, 53 (46.1 %) represented Supervision without Quotas, 24 (20.9 %) Comprehensive Quota-Oriented and 13 (11.3 %) the Unsupervised Volunteer models. The mean age of the CWs engaged in the different models was significantly different with the mean age of CWs of Direct Dealing model being the lowest (43.6 ± 1.5 years) and those of the Comprehensive Quotas-Oriented model being the highest at 52.3 ± 1.5 years (*p* = 0.024). The level of education and marital status of CWs engaged in the different models were also significantly different (p < 0.05). Compared with the CWs of other models, a significantly higher proportion of the CWs of the ‘Comprehensive Quotas-Oriented model also worked as treatment supporters and tracked TB patients lost to follow up (*p* < 0.001) in addition to referral of presumptive TB cases. Also all (100 %) of the CWs of Comprehensive Quotas-Oriented model had good knowledge of TB symptoms identification compared with 60, 67.9 and 53.8 % of CWs of Direct Dealing, Supervision without Quotas and Unsupervised Volunteer model respectively (*p* = 0.004).

### Comparative impact on TB testing and TB case detection

CWs referred 4–22 % of clients TB tested, and 4–24 % of the total TB cases detected. Of the 2282 TB cases notified in the study areas, CWs contributed 353 (15.5 %). The proportion of TB investigation due to CW referral was 20.8–24.4 % for Comprehensive Quotas-Oriented, Direct Dealing and Supervision without Quotas model while the Unsupervised Volunteer model contributed 3.9 % to TB investigation. Overall, an average of 5 presumptive TB clients was referred for TB investigation for every TB case detected.

### Performance per community worker

The overall median presumptive TB client referral for the CWs of all models was 10.5 (IQR 4.0, 33.8) per year and the mean TB case contributed per CW per year was 2.8. For the CWs of Comprehensive Quota Oriented model, the median presumptive TB case referral was 48.0 (IQR 42.8, 58.8) and the mean TB case contributed was 7.1 per CW per year. However the median presumptive TB case referral and the mean TB case contributed by CWs of other model per year ranged between 3.0–13.0 (IQR 1,18.5) and 0.53–2.5 respectively. The referrals per TB case reported by the CWs of Quotas Oriented and Direct Dealing models was 6 while that of the CWs of Unsupervised Volunteer and Supervision Without Quotas models was 3.1 and 3.3 respectively.

The models offered CWs a wide range of annual compensation of US $ 7.5–US $34.3 per presumptive TB client referred and US $45.2–US $106.7 per TB case detected. The annual compensation per CW was highest ($320) for the Comprehensive Quotas-Oriented model and lowest ($80) for the Unsupervised Volunteer model. However, the CWs compensation per TB patient diagnosed was lowest for the Comprehensive Quotas-Oriented model ($45), compared with $64, $75 and $107 for the, Supervision without Quotas, Direct Dealing and Unsupervised Volunteer model respectively (Table 3).

The median time spent on finding presumptive TB cases per week was longest for CWs of the Comprehensive Quotas-Oriented model at 37.5 h (IQR 35.0–46.8 h) compared 8.0 h (IQR 6.0–10.5), 6.0 h (IQR 3.0–9.0) and 2.0 h (IQR 2.0–2.5) for the CWs of Direct Dealing, Supervision without Quotas and Unsupervised Volunteer models respectively (H = 69.14, *p* < 0.001).

### Predictors of CW performance

Table 4 shows that CWs who were above 50 years, worked as treatment supporter and had good knowledge of symptoms and signs of TB referred a significantly higher number of presumptive TB cases than those who were below 50 years, never worked as treatment supporter and had poor knowledge of symptoms and signs of TB. Although age of the CWs, the period of their engagement as CW for TB, hours spent on presumptive TB referral per week, compensations received and knowledge of TB symptoms were significantly correlated with presumptive referral (*p* < 0.001), hours spent on presumptive referral per week had the highest correlation coefficient (0.56).

**Table 4:** Factors affecting presumptive TB referrals by community workers

Variable	n	Median	IQR	U	P
Age group					
Less than 50 years	Less than 50 years	6.50	1.0–21.8	1177.5	0.010
50 years and above	51	14.0	9.0–40.0		
Gender					
Male	42	12.0	4.8–22.0	1466.0	0.697
Females	73	10.0	2.0–41.0		
Worked as Treatment supporter					
Yes	42	38.5	17.5–48.5	366.5	<0.001
No	73	6.0	1.0–12.5		
Participation in other programs					
Yes	99	11.0	3.0–37.0	641	0.221
No	16	8.0	1.3–17.5		
Knowledge of suspect identification					
Poor	33	9.0	1.0–13.5	943.0	0.011
Good	82	14.0	4.0–42.0		

Note: *IQR* interquartile range

*U* Mann Whitney *U* test

Multiple regression was used to assess the ability of hours spent on presumptive TB referral weekly, the period of engagement as CW for TB, compensation received and model to predict presumptive TB referral. Hours spent weekly on presumptive TB referral made the strongest unique contribution (Beta = 0.514, *P* < 0.001) to explaining presumptive TB referral (the dependent variable) when all the variance explained by all other variables in the model was controlled for.

## Discussion

### Model influences on performance

The mode and criteria for CW selection, supervision and compensation for the models studies varied. Supervision, compensation, training and monitoring and evaluation mechanisms appear to have influenced CWs performance. However, the time spent weekly on presumptive TB referral made the strongest unique contribution (Beta 0.514, *p* < 0.001) to explaining presumptive TB referral in this study. The time one has to volunteer is generally determined by one’s income source [14]. A possible explanation for the motivation of the CWs of the Comprehensive Quota-Oriented model to dedicate more time on presumptive TB referral could be the combination of high monthly payment and a quota of 5 referrals per month. The CWs under this model were also involved in supervision and tracing of TB patients, so they had greater access to people at high risk since they visit TB affected households.

Most countries lack a coherent national policy on motivation strategies for CWs. Experience from large scale sustainable CWs programmes from Nepal, Bangladesh, India, Ethiopia and Iran showed that a variety of approaches to remuneration with varying degree of success [15–21]. Evidence tends to show that CWs work best when volunteering is motivated by social status, community appreciation, or common good (social market theory) or when CWs are trained work full time and are remunerated based on hours expected of them (monetary market) [22, 23]. Anticipated compensation is correlated with productivity [24, 25]. However, inadequate training, poor supervision, irregular incentives and lack of acceptance or appreciation by the community could demotivate CWs whether they are volunteers or fully paid. On the contrary, the use of incentives or low remuneration (mixed methods) could lead to discontent or shift the focus of the CWs to the incentive [14]. This may explain why the performance of CWs of the Unsupervised Volunteer model was poor compared with CWs of other models. It also explains why (during the focus group discussion) virtually all the CWs of the four models complained that the incentive was poor but were motivated to work because of the respect and recognition they got from the community.

### Individual influences on performance

In this study, age, length of work as CWs, and knowledge of TB symptoms were associated with increased referral of presumptive TB cases. CWs above 50 years referred more presumptive TB than younger ones. The reasons why older CWs were more effective referring symptomatic community members is unknown. One possibility is that older community health workers may have social networks comprised of older people, who are at higher risk of TB in Nigeria. Another possibility is that compared to younger CWs, older CWs may command more authority to compel community members to seek care and they may have more spare time to dedicate to seeking symptomatic community members. There is possibility that the longer CWs are involved in volunteering work, they more likely they acquire more skills of identifying presumptive TB case which will ultimately result in increase presumptive TB case referral.

### Local factors influencing performance

As with all enhanced case-finding programs, CWs model effectiveness was modulated by the performance of the overall TB program [26]. CWs contributed between 3.9 and 24.2 % of TB presumptive cases investigated and 4.4–22.9 % of TB cases notified. Despite the high volume of referred presumptive TB cases in the Comprehensive Quotas-Oriented model the proportion of case diagnoses was not the highest in this model. This could be because it was embedded in a well-functioning passive case-finding TB program and the monthly quota of 5 given to the CWs. The models were implemented on varying scales, so model performance is best assessed on a per CW basis not according to overall impact on LGA case detection.

### National factors influencing performance

CWs referred an average of 4.7 (range 3–6) presumptive clients for every TB case detected. This ratio is much lower than in other countries [10, 27] suggesting a very highly specific screening algorithm. Traditionally a ratio of 10 referrals per TB case detected is considered appropriate [16, 27]. All the models may have underperformed compared with other countries because of the 2–3 week chronic cough screen promoted in the National CTBC DOTS curriculum as the sentinel sign for referral. This algorithm is capable of identifying only 65 % of Nigerian TB [5]. Presumptive cases are then usually tested with smear microscopy reducing the actual yield of the CTBC algorithm to less than 40 % of all TB, too limited.to make a major impact on case detection [7, 16]. An effective community active case finding program in Nigeria would need to significantly expand the volume of people referred and the sensitivity of the diagnostic algorithm. However this presupposes that the health system is able to receive and process such a high volume of samples. The future of high volume CTBC in Nigeria may be sputum sample pooling to facilitate deployment of tools such as Xpert MTB/Rif in a cost effective manner [5].

To appreciate the impact of the CW models, it is also important to understand the socio-cultural, economic, political and the health system context in which CWs interventions operate [15, 28–32]. Countries like Ethiopia, Iran and Nepal [15, 21, 33] have successful, high coverage outreach programs because of high levels of government commitment and investment in an effective CWs workforce. This might not be true in Nigeria as the CTBC programs are primarily sponsored by implementing partners with varying degree of government engagement. At present the CTBC program is not sustainable judging from the poor funding of the health care sector in the Nigeria. In 2013, only 5.7 % of the country’s government budget was spent on health which was much lower than the expected 15 % [34].

Although studies have shown that involving CWs generally can be affordable and cost effective [35–38]. There is need for further research to compare the cost of DOTs management of TB and community treatment of TB in the country. There is no doubt that for CTBC to be successful in Nigeria there is need for a strong political will, proper selection of the right people who are community minded, well- trained and motivated.

### Limitations

This study was limited in size and there was significant covariance among elements of the models which made it difficult to discern the effects of individual program elements. The way that the programs were designed often made it difficult to determine the proportion of presumptive referrals of the CWs that actually reached the health facility and were investigated for TB. Some programs neglected to capture figures on the total pool of community members screened for TB symptoms. The number of presumptive referrals recorded on the CWs records was assumed to reflect the number of presumptive referrals that got to the health facilities since the figure was generally very low for the CWs of all the models. In some cases, the data on presumptive referral of CWs collected from the records of the CBOs matched the data from the records of the state TB program. Where possible, the data from the TB program were used for analysis to minimize bias. Our study did not include administrative, indirect, training and other costs associated with the various models, and so we cannot determine the true cost per case, but rather the CW compensation per case.

## Conclusion

This study compared four models of community enhanced case finding and found they all contributed relative little to addressing Nigeria’s high burden of undiagnosed TB. There is an urgent need to consider not only the design, but also the implementation and the setting in which these models operate in order to improve their contribution. If well organised, community-based TB care could be a core strategy of Nigerian TB control as 75 % people with smear-positive TB are symptomatic and therefore potentially recognizable for well-trained community members. A highly supervised and incentivized model that leverages treatment support roles, and effective CW training and supervision should be considered when building future TB community programs. The TB case detection crisis in Nigeria requires an evidence-based community case finding strategy [5]. Given the right tools and working conditions, CWs can contribute significantly to TB case detection in Nigeria.
